# WISDOM OF THE ELDERS, VOICES FROM OUR AMERICA

**DOI:** 10.1093/geroni/igz038.2104

**Published:** 2019-11-08

**Authors:** Ifeoma Nwankwo

**Affiliations:** Vanderbilt University, Nashville, Tennessee, United States

## Abstract

The fundamental goal of WOE was to determine whether and how autobiography production can enhance and provide valuable data about African American seniors’ mental health, past and present. The second goal of WOE was to determine whether and how project partners could use the findings from WOE, particularly those about resilience and strategies for coping with challenging situations, as the basis for new interventions, studies, publications, curricula, and educational programs for learners across contexts, generations, levels, and disciplines. Outcomes are illustrated by rich examples of the genesis, history, products and pedagogy of the project.

